# Chlorophenyl thiophene silicon phthalocyanine: Synthesis, two-photon bioimaging-guided lysosome target, and *in vitro* photodynamic efficacy

**DOI:** 10.3389/fphar.2023.1168393

**Published:** 2023-04-13

**Authors:** Le Xu, Tiantian Zhang, Bingcheng Huang, Fangmei Zheng, Yan Huang, Yuyang Li, Yiru Peng, Linying Chen

**Affiliations:** ^1^ College of Chemistry and Materials, Fujian Provincial Key Laboratory of Advanced Materials Oriented Chemical Engineering, Fujian Provincial Key Laboratory of Polymer Materials, Fujian Normal University, Fuzhou, China; ^2^ Department of Pathology, The First Affiliated Hospital of Fujian Medical University, Fuzhou, Fujian, China

**Keywords:** chlorophenyl thiophene, silicon phthalocyanine, cell imaging, photodynamic therapy, apoptosis, lysosome-specific target

## Abstract

The development of efficient photosensitizers with high singlet oxygen quantum yield, strong fluorescent emission, excellent photostability, and specific organelle targeting is in great demand for the enhancement of PDT treatment efficiency. This study designed and synthesized a new two-photon photosensitizer chlorophenyl thiophene axially substituted silicon (IV) phthalocyanine (CBT-SiPc). CBT-SiPc showed specific targeting of lysosomes in living cells and good biocompatibility. Furthermore, high ^1^O_2_ generation efficiency and high PDT efficiency in MCF-7 breast cancers under irradiation were also demonstrated. The novel CBT-SiPc showed great potential in the application of lysosome-targeted and two-photon bioimaging-guided photodynamic cancer therapy.

## Graphical Abstract

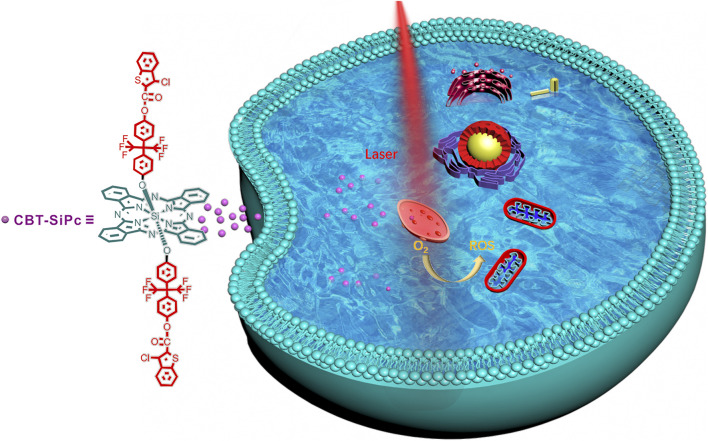

## 1 Introduction

Photodynamic therapy (PDT) has attracted attention for its low dark toxicity and reduced side effects, controllability, and high cure accuracy (D'Alessandro and Priefer, 2020; Simoes et al., 2020). Phthalocyanines (Pcs) and their derivatives are promising second-generation photosensitizers owing to their extraordinary properties such as strong absorption in the near-infrared region, photostability, and high ROS quantum yield (Li et al., 2019; Galstyan, 2021). Silicon phthalocyanines with two axial substitutions reduce aggregation in solution and can be synthetically designed, thereby creating wide scope for modulation of their optical, chemical, and electronic properties (Li et al., 2019). However, their drawbacks include the easy formation of aggregates in aqueous solutions, leading to the low production of reactive oxygen species (ROS) and a lacking of targeting of cancer cells (Darwish, 2020). Many efforts have been focused on improving the PDT efficacies of Pcs by inducing various substitutions to their axial/peripheral positions (Chen Y. et al., 2018; Almeida-Marrero et al., 2021).

PDT efficacy has recently been shown to be associated with the amount of cancer cell uptake and subcellular localization of photosensitizers (Ming et al., 2021). Because the ROS lifetime is short and the action radius is limited (Luo et al., 2021), only photosensitizers proximal to the organelle are directly affected by PDT (Darwish, 2020).

Most PSs are preferentially localized in the plasma membrane (Kim et al., 2014), Golgi apparatus (Soriano et al., 2014), endoplasmic reticulum (Yu et al., 2018), nucleus (Gao and Lo, 2018), mitochondria (Valli et al., 2019), and lysosomes (Miretti et al., 2021). The most common PDT strategies are photo-damage of the mitochondria and lysosomes. In particular, lysosomes have attracted attention because they are involved in the maintenance of cellular homeostasis and regulation or interaction with other organelles (Liu et al., 2010; Zou et al., 2017; Chen et al., 2019). The induction of cancer cell programmed necrosis by nano-drugs with lysosome-targeting capability has been reported and has become a hot topic for overcoming cancer resistance to apoptosis and therapy (Yu et al., 2012; Meng et al., 2019). Lysosome-dependent cell death (LDCD) exploits lysosomal membrane permeabilization (LMP) to translocate lysosomal contents to the cytoplasm and then executes tumor cell death (Wang et al., 2014; Ding et al., 2018).

Bioactive molecules including benzothiophene derivatives have been widely used in agrochemical, medicinal agent, and chemical sensor applications (Elkanzi, 2018; Agoni et al., 2020; Mishra et al., 2020). Moreover, some benzothiophene derivatives can label organelles to improve PDT efficacy. For example, benzo[b]thiophene substituted porphyrin accumulated in the mitochondria and nucleus and damaged the function of these organelles to induce the death of MCF-7 breast cancer cells (Mohareb et al., 2016).

This study prepared a novel di-((3-chlorophenylthiophene-2-ester)hexafluorophenoxy) axial substituted silicon phthalocyanine (CBT-SiPc). Thanks to the chlorophenyl thiophene groups, CBT-SiPc showed hindrance that reduced the aggregation of silicon phthalocyanines to some extent and showed specific lysosome targeting. The heavy atom S in the chlorophenyl thiophene groups also promoted intersystem crossing (ISC), which increased the ability to produce ROS for silicon phthalocyanine (Cai et al., 2017; Bai et al., 2021). We also studied the structures, photophysical and photochemical properties, organelle targeting ability, and *in vitro* photodynamic efficacy of CBT-SiPc.

## 2 Experimental section

### 2.1 Materials and equipment

3-Chloro-benzo[b]thiophene-2-carboxylic acid, hexafluoro bisphenol A, 4-dimethylaminopyridine (DMAP), and 1-ethyl-3-(3-dimethylaminopropyl) carbodiimide hydrochloride (EDC·HCl) were supplied by Energy Chemical Company (Shanghai, China). 2,7-Dichlorodihydrofluorescein diacetate (DCFH-DA), LysoTracker green, and the Apoptosis Detection Kit with Annexin V-FITC and propidium iodide were purchased from Beyotime Biotechnology (Shanghai, China). Cell Counting Kit-8 (CCK-8) was obtained from GlpBio Biotechnology Co. (Shanghai, China). Dichloride silicon phthalocyanine (SiPcCl_2_) and unsubstituted zinc (II) phthalocyanine (n-ZnPc) were prepared as we described previously (Ding et al., 2013). Phosphate-buffered saline (PBS), fetal bovine serum (FBS), and Dulbecco’s minimum essential media (DMEM) were obtained from Gibco Life Technologies (USA). MCF-7 breast cancer cells were supplied by Shanghai Kefeng Biological Technology Co. (Shanghai, China). Other chemicals were purchased from Sinopharm Chemical Reagent Co. (Shanghai, China).

A Perkin Elmer spectrometer was used to measure the infrared radiation (IR) spectra. 1 H nuclear magnetic resonance (NMR) spectra were recorded on a Varian Unity-400 NMR spectrometer. Mass spectra were recorded on a Bruker mass spectrometer. UV-Vis was recorded on a Cary 50 UV-Vis spectrophotometer, and fluorescence spectra were measured on an FL900/FS920 steady-state fluorescence spectrometer. High-performance liquid chromatography (HPLC) (SHIMADZU Essentia LC-16P) was used to analyze the CBT purity using acetonitrile: water (v/v = 40:60) as the mobile phase and a Zorbax SB-Aq C18 (4.6 × 250 mm, 5 μm) reversed-phase column as the solid phase at 25°C with a flow rate of 1 mL/min. High-resolution mass spectrometry was measured on an Agilent 6550 iFunnel Q-TOF LC/MS System (Japan).

### 2.2 Synthesis of 3-chloro-benzothiophene-2-ester hexafluorophenol (CBT-OH)

3-Chloro-benzo[b]thiophene-2-carboxylic acid (0.43 g, 2.00 mmol), hexafluoro bisphenol A (0.67 g, 2.00 mmol), DMAP (70.00 mg, 0.6 mmol), EDC HCl (0.50 g, 2.60 mmol), and dichloromethane (CH_2_Cl_2_) (40.00 mL) were refluxed at room temperature for 24 h with monitoring of the reaction by thin layer chromatography (TLC). The reacted mixture was washed with water. The collected organic phase was dried with anhydrous MgSO_4_ and then filtered. The organic phase was collected, and the organic solvent was evaporated under reduced pressure to obtain the crude solid product. The crude product was further purified on a silica gel chromatography column using CH_2_Cl_2_/ethanol (v/v = 5:1) as eluent twice. A white solid with a yield of 38.6% was obtained, with the following characteristics: IR/cm^−1^: 3446, 1610, 1499, 1192, 1059, 958, 926, 802, 744, 698, 586; ^1^H NMR (CDCl_3_, 400 MHz, δ/ppm): 5.21 (s, 1H; H^1^), 12.16–12.18 (m, 2H; H^2^), 12.47–12.49 (d, *J* = 8 Hz, 2H; H^4^), 12.71–12.80 (m, 4H; H^3^), 12.87–12.96 (m, 2H; H^6^), 13.28–13.37 (m, 2H; H^5^). ESI-MS for C_24_H_13_ClF_6_O_3_S (m/z): 571. Found: 571.58 [M + K]^+^.

### 2.3 Synthesis of bis-((3-chlorobenzothiophen-2-ester)hexafluorophenoxy) axially substituted silicon phthalocyanine (CBT-SiPc)

A mixture of CBT-OH (0.12 g, 0.20 mmol), SiPcCl_2_ (0.06 g, 0.10 mmol), anhydrous K_2_CO_3_ (0.69 g, 5.00 mmol), and toluene (40 mL) was added to a 100-mL flask and stirred at 140°C for 48 h. with monitoring by TLC. After being cooled to room temperature, the reaction mixture was filtrated, and the filtrate was evaporated under reduced pressure. The crude product obtained was separated and purified on a silica gel chromatography column using CH_2_Cl_2_ as an eluent, and a blue solid was collected. Yield: 30%, with the following characteristics: IR/cm^−1^: 1741, 1504, 1272, 1176, 1081, 1045, 928, 872, 738.^1^H NMR (CDCl_3_, 400 MHz, δ/ppm): 5.61–5.63 (m, 4H; H^5^), 6.52–6.58 (m, 4H; H^1^), 6.77–6.81 (m, 4H; H^2^), 7.18–7.35 (m, 4H; H^3^), 7.60–7.65 (m, 4H, H^4^), 7.89–7.96 (m, 2H; H^6^), 8.10–8.12 (m, 2H; H^7^), 8.38–8.42 (m, 8H; H^8^), 9.63–9.67 (m, 8H; H^9^). ESI-MS for C_80_H_44_Cl_2_F_12_N_8_O_6_S_2_Si (m/z): 1599. Found: 1599.89 [M + H]^+^. Calculated percentages of C_80_H_44_Cl_2_F_12_N_8_O_6_S_2_Si: C 60; H 2.75; N 7; S 4; found: C 61.99; H 4.23; N 6.98; S 4.23.

### 2.4 Cell uptake of CBT-SiPc in MCF-7 breast cancer cells

MCF-7 breast cancer cells were cultured in DMEM containing 10% FBS and 1% penicillin–streptomycin at 37°C with 5% CO_2_. CBT-SiPc was dissolved in DMSO to make a stock solution (1.0 mM). The cells were seeded into 35 mm confocal dishes at a density of 1.0 × 10^4^ cells per dish for 24 h and then treated with CBT-SiPc-containing medium (3.0 μM) for 10 h. Next, the medium was removed and the cells were washed twice with PBS. Fluorescence images of CBT-SiPc in MCF-7 breast cancer cells were obtained using a confocal laser scanning microscope (CLSM).

### 2.5 Subcellular localization

The MCF-7 breast cancer cells were seeded into 35 mm confocal dishes at a density of 1.0 × 10^4^ cells per dish for 24 h and then treated with CBT-SiPc-containing medium (3.0 μM) for 10 h. After incubation, the medium was removed and the cells were washed twice with PBS. LysoTracker Green (50 nm) was then added to dishes to stain the lysosomes of the cells for 30 min before rinsing three times with PBS. The experimental procedure was similar to that described previously (Forteath et al., 2012).

### 2.6 Intracellular ROS generation

DCFH-DA was used as the probe to determine the intracellular ROS generation ability in MCF-7 cells (Zhang et al., 2014; Karaoğlu et al., 2018). MCF-7 cells were seeded in four-well plates at a density of 1.0 × 10^4^ cells per dish for 24 h. The cells were co-incubated with CBT-SiPc (3.0 μM) for 10 h. After that, the cells were irradiated with a 671 nm laser (100 mW/cm^2^) for 10 min and then treated with DCFH-DA for 1 h at 37°C. Next, the medium was removed and the cells were washed three times with PBS. Finally, the production ability of ROS was visualized by CLSM.

### 2.7 Annexin V-FITC/PI flow cytometry

The MCF-7 breast cancer cells were seeded into 35 mm confocal dishes at the density of 1.0 × 10^4^ cells per dish for 24 h and then treated with CBT-SiPc-containing medium (3.0 μM) for 10 h. After incubation, the cells were exposed to laser irradiation (671 nm, 100 mW/cm^2^) for 0, 5, 10, 15, and 20 min, respectively. Then, the cells were observed by CLSM.

The apoptosis/necrosis mechanism of CBT-SiPc-mediated photodynamic therapy was detected by flow cytometry analysis with double staining using an Annexin V-FITC Apoptosis Detection Kit (Meng et al., 2019). MCF-7 cells were seeded in four-well plates (1.0 × 10^4^ cells/well, 24 h) and subsequently incubated with CBT-SiPc (3.0 μM) for 10 h. The blank group was directly cultured in fresh DMEM medium instead of the old medium for 10 h. After treatment, the cells were exposed to a 671 nm laser (100 mW/cm^2^) for 10 min and then incubated for another 4 h. The resulting cells were stained with the mixture of Annexin V-FITC (5 µL) and propidium iodide (PI) (10 µL) for 15 min, and the mixture was analyzed with flow cytometry to assess the number of apoptotic cells. All experiments were performed in triplicate.

### 2.8 Photodynamic efficacy of CBT-SiPc against MCF-7 cells

The photodynamic therapy efficacy of CBT-SiPc against MCF-7 cells was determined by CCK-8 assay (Alshammari et al., 2020). MCF-7 cells were cultured in a cell culture medium containing 1% penicillin, streptomycin, and 10% bovine serum albumin at 37°C with 5% CO_2_. MCF-7 cells with a density of 1 × 10^4^ cells per well were cultured in 96-well plates with 100 μL of culture medium for 24 h. The cells were then treated with various concentrations of CBT-SiPc (0, 1, 2, 3, 4, and 5 μM) for 24 h. Next, the cells were exposed to the absence and presence of laser (671 nm, 100 mW/cm^2^, 10 min). Finally, the surviving cells were measured by CCK-8. The relative cell viability was calculated using Eq. 5 (Özdemir et al., 2020).

$$Relative\;cell\;viability\;\left(\%\right)=\frac{{OD}_{sample}-{OD}_{blank}}{{OD}_{control}-{OD}_{blank}}\times 100$$ (1)

## 3 Results and discussion

### 3.1 Synthesis and characterization

**FIGURE 1:**
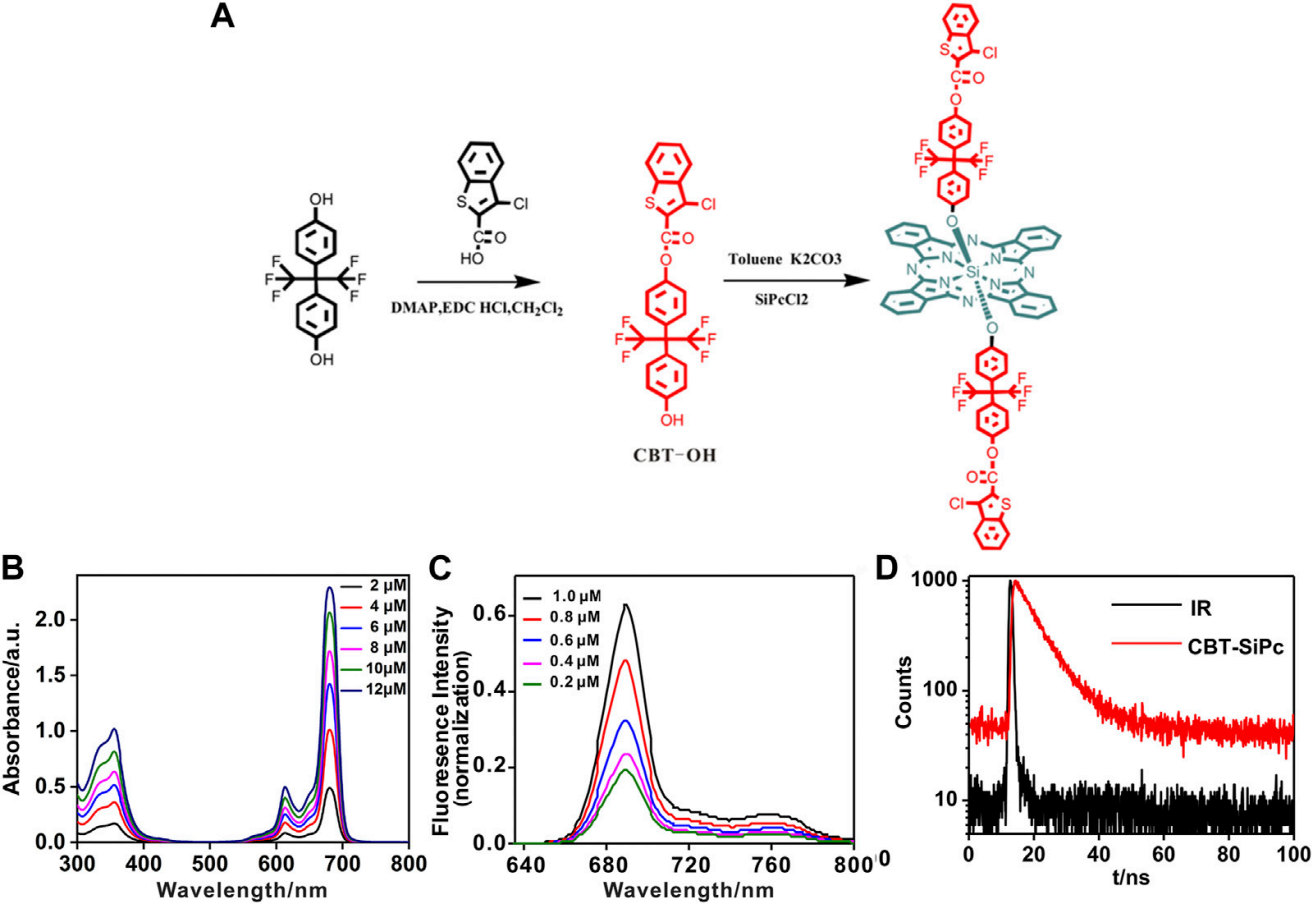
**(A)** Synthesis scheme for chlorophenyl thiophene silicon (IV) phthalocyanine (CBT-SiPc). **(B)** UV/Vis spectra for different concentrations of CBT-SiPc. **(C)** Fluorescence spectra of different concentrations of CBT-SiPc (*λ*_ex_ = 615 nm). **(D)** Fluorescence decay curves of CBT-SiPc in DMF. (*λ*_ex_ = 405 nm, C = 10 uM).

The scheme for the synthesis of chlorophenyl thiophene silicon phthalocyanine (CBT-SiPc) is shown in Figure 1A. 3-Chloro-benzoaryl thiophene-2-carboxylic acid reacted with hexafluoro bisphenol A to produce the precursor CBT-OH. CBT-OH coupled with silicon phthalocyanine dichloride (SiPcCl_2_) *via* nucleophilic substitution to obtain CBT-SiPc. CBT-SiPc was soluble in common organic solvents such as dimethyl sulfoxide (DMSO), N, N-dimethylformamide (DMF), and tetrahydrofuran (THF). The structures of CBT-OH and CBT-SiPc were confirmed by ^1^H NMR, IR, ESI-MS, and HRMS.

The CBT-SiPc purity was confirmed by ^1^H NMR, HPLC, and HRMS spectra. Resonances at 9.63 and 8.38 ppm were assigned to signals of the phthalocyanine ring with 16 protons. The three resonances at 5.61–5.63, 7.89–7.96, and 8.10–8.12 were designated to the six aromatic protons of chlorophenyl thiophene, while the four sets of resonances at 6.52–6.58, 6.77–6.81, 7.18–7.35, and 7.60–7.65 were ascribed to the aromatic protons of the hexafluoro bisphenoxy groups (Supplementary Figure S5). The ESI-MS and HRMS spectra of CBT-SiPc showed an intense singlet-charged molecular ion peak (Supplementary Figures S6, S7), which conformed to the corresponding proposed structure. The CBT-SiPc purity was further confirmed by HPLC. Only one structure isomer peak was observed for CBT-SiPc, with a retention time of 3.00 min (Supplementary Figure S11).

### 3.2 Photophysical and photochemical properties of CBT-SiPc

The UV/Vis spectra of CBT-SiPc in DMF are shown in Figure 1B. CBT-SiPc exhibited typical spectra for phthalocyanine with a B band at about 356 nm and a Q band at about 680 nm, which is redshifted compared to the spectra for SiPcCl_2_ (Davies et al., 2012). The Q band intensity rose with increasing CBT-SiPc concentration, indicating that CBT-SiPc mainly existed as a monomer in DMF. This could be attributed to the steric hindrance effect caused by CBT groups in the axial position of silicon phthalocyanines, which reduced CBT-SiPc aggregation to some extent.

The fluorescence spectra of CBT-SiPc in DMF are shown in Figure 1C and Supplementary Figure S9. The fluorescence decay curve is shown in Figure 1D. Upon excitation at 610 nm, the maximum emission of CBT-SiPc was located at 689 nm, and the fluorescence intensities rose with increasing CBT-SiPc concentration. The fluorescence quantum yield (*Φ*_*F*_), fluorescence lifetime (*τ*_*s*_), and singlet oxygen quantum yield (*Φ*_*Δ*_) of CBT-SiPc were measured according to the literature (Chen X. et al., 2018). The fluorescence quantum yield of CBT-SiPc was 0.121, the calculated fluorescence lifetime was 7.26 ns (Figure 1D), and the singlet oxygen quantum yield (*Φ*_*Δ*_) was 0.30 (Supplementary Figure S9).

### 3.3 Lysosome colocalization assay in MCF-7 breast cancer cells

**FIGURE 2:**
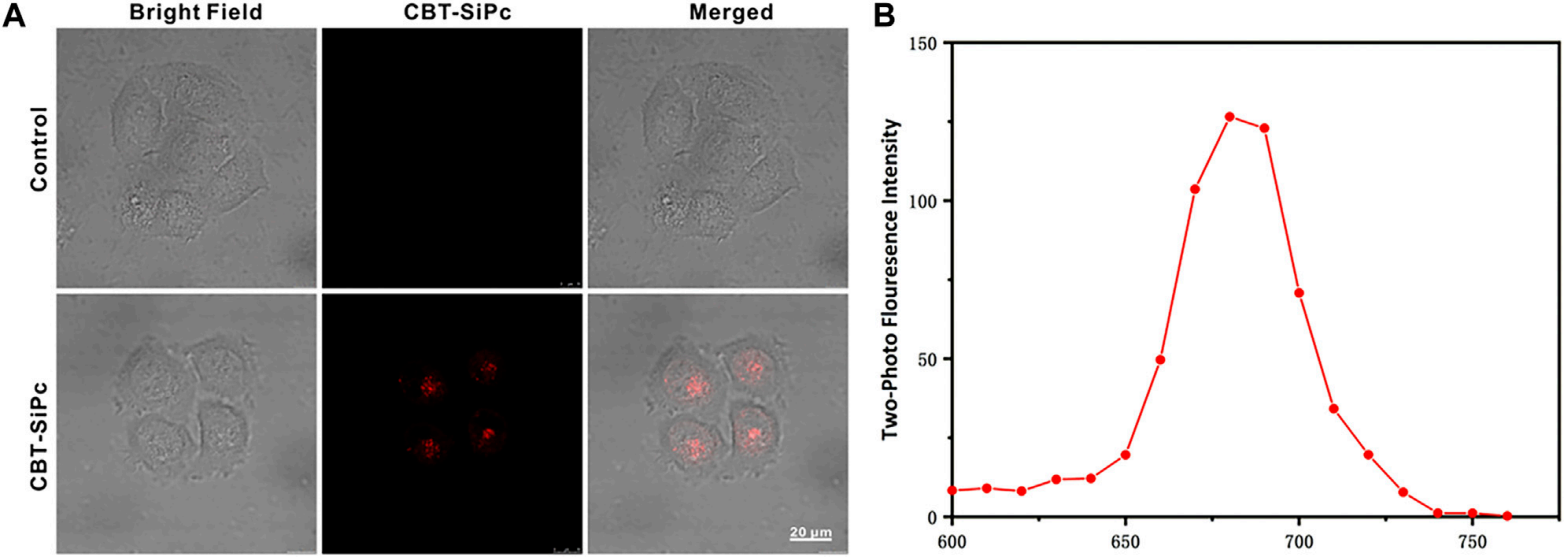
Two-photon fluorescence images of CBT-SiPc in MCF-7 breast cancer cells. **(A)** Two-photon fluorescence images of CBT-SiPc in MCF-7 breast cancer cells (2 μM, red fluorescence, excited by 860 nm fs laser. The fluorescence was monitored at 650–700 nm). **(B)** Confocal lambda scan spectra of CBT-SiPc in MCF-7 breast cancer cells excited by an 860 nm fs laser.

The cellular uptake of CBT-SiPc by MCF-7 cells was visualized through two-photon fluorescence imaging (Figure 2A). After incubation with CBT-SiPc for 24 h (Supplementary Figure S10), the cells showed strong red fluorescence upon excitation by 860 nm fs laser, indicating the good biocompatibility of CBT-SiPc. The confocal lambda scan spectrum of the red fluorescence was consistent with the fluorescence spectra of CBT-SiPc in solution, which demonstrated that the red fluorescence belonged to CBT-SiPc (Figure 2B).

**FIGURE 3:**
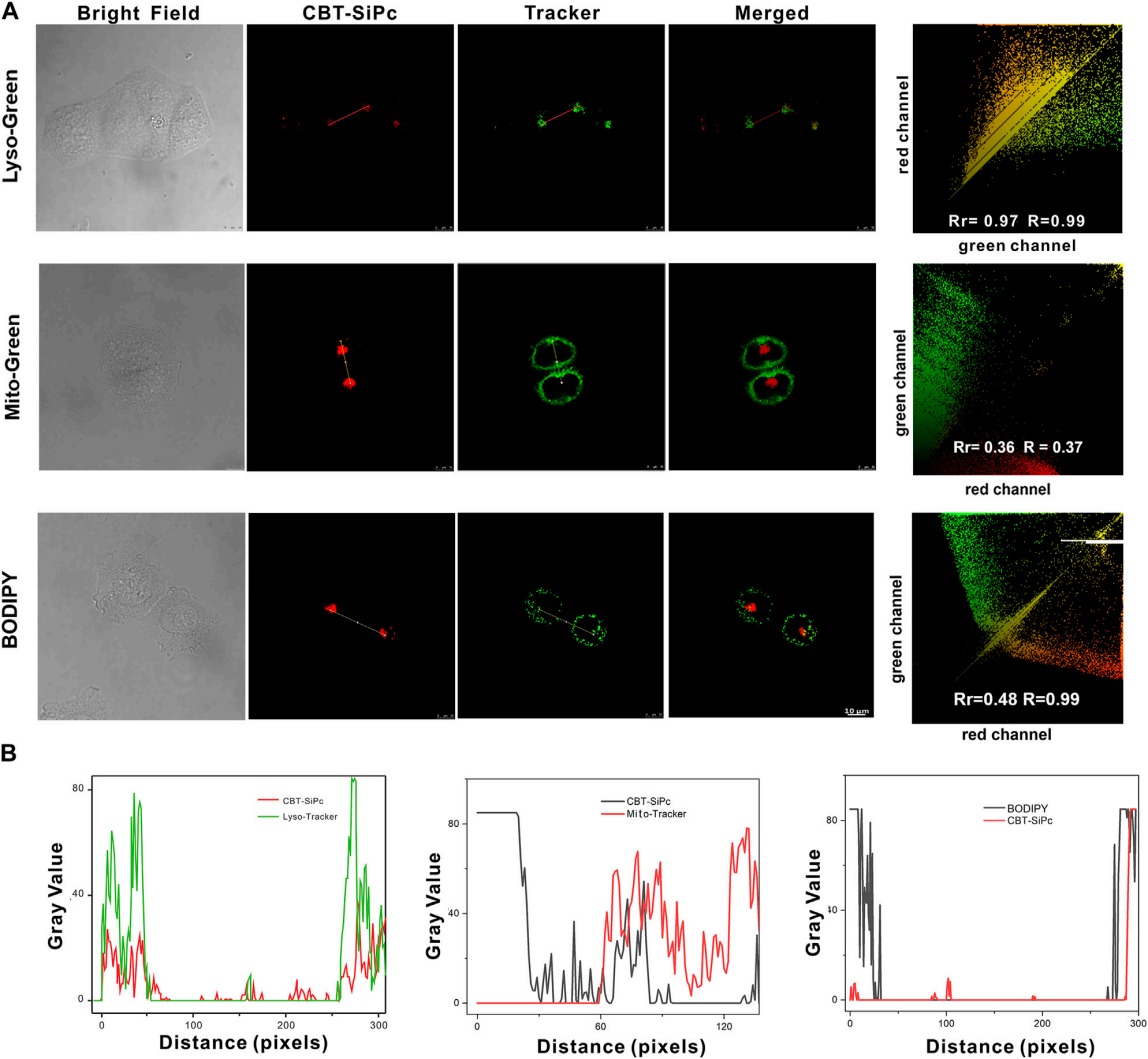
Subcellular localization of CBT-SiPc in MCF-7 breast cancer cells. **(A)** Intracellular distribution of CBT-SiPc in MCF-7 cells. Lysosomes, mitochondria, and lipid droplets were stained with commercial LysoTracker (Lyso-Green), MitoTracker (Mito-Green), LipidTracker (BODIPY), and merged images observed by CLSM; **(B)** co-localization curves of CBT-SiPc with LysoTracker (Lyso-Green), MitoTracker (Mito-Green), and LipidTracker (BODIPY) (CBT-SiPc, excited by 860 nm fs laser). The fluorescence was monitored at 650–750 nm. Lyso-Green was excited at 552 nm, and the fluorescence was monitored at 580–620 nm. Mito-Green was excited at 488 nm, and the fluorescence was monitored at 500–530 nm. BODIPY was excited at 488 nm, and the fluorescence was monitored at 490–590 nm.

Two-photon confocal laser scanning microscopy was used to investigate the intracellular distribution of the CBT-SiPc in MCF-7 cells. Commercial LysoTracker (LysoTracker Green), MitoTracker, and lipid droplet trackers BODIPY were incubated with CBT-SiPc, respectively, to assess organelles targeting by CBT-SiPc through two channels. As shown in Figure 3A, the red fluorescence was from CBT-SiPc, and the green fluorescence from the commercial LysoTracker Green, MitoTracker, and lipid droplet trackers BODIPY, respectively. The Pearson’s correlation coefficients were 0.97, 0.36, and 0.48, respectively. These results indicated that CBT-SiPc perfectly overlapped with LysoTracker. The co-localization curves of CBT-SiPc with LysoTracker and MitoTracker further confirmed these results (Figure 3B). The Mander’s overlap coefficient for CBT-SiPc with LysoTracker was 0.99. The CBT-SiPc targeting of lysosomes could be explained by the interaction of the S atoms of the CBT groups with the protein residues containing S atoms through S–S intermolecular interactions (Gleiter et al., 2018; Brilkina et al., 2019; Liu et al., 2021; Liu et al., 2022).

### 3.4 *In vitro* photodynamic therapy efficacies of CBT-SiPc against MCF-7 cells

**FIGURE 4:**
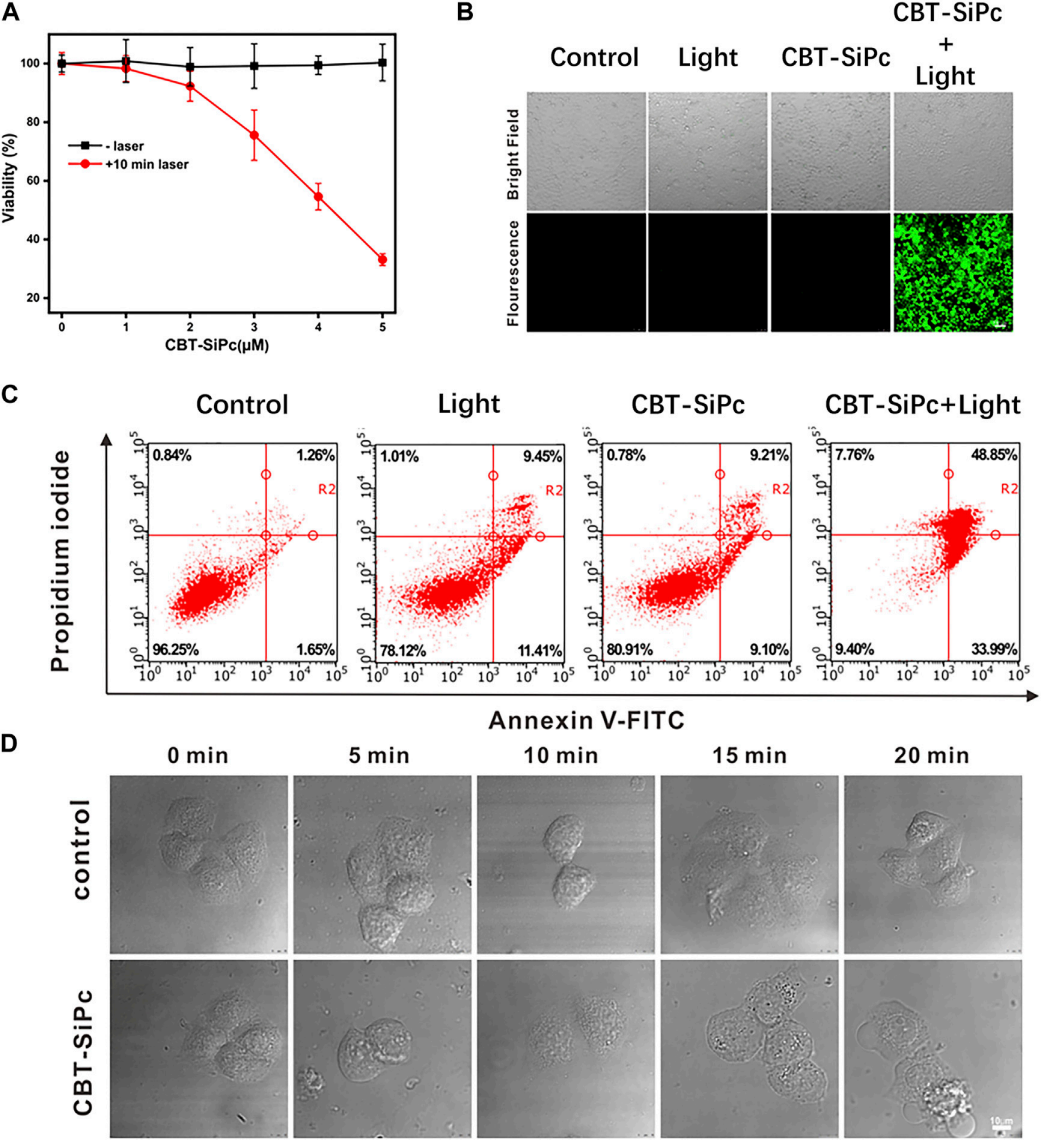
*In vitro* photodynamic therapy efficacy of CBT-SiPc against MCF-7 breast cancer cells. **(A)** Concentration-dependent phototoxicity of CBT-SiPc against MCF-7 cells before and after irradiation (671 nm, 100 mW/cm^2^, 10 min). **(B)** Confocal fluorescence images of intracellular ROS generation by CBT-SiPc (671 nm, 100 mW/cm^2^, 10 min) in MCF-7 cells before and after irradiation using CM-H_2_DCFDA as the probe (*λ*_ex_ = 488 nm; *λ*_em_ = 490–590 nm). **(C)** Flow cytometry analysis of CBT-SiPc-mediated, PDT-induced MCF-7 cells apoptosis/necrosis mechanism before and after irradiation (671 nm, 100 mW/cm^2^, 10 min). **(D)** Morphological changes of MCF-7 cells (co-incubated with CBT-SiPc) by confocal microscopy upon laser irradiation (671 nm, 100 mW/cm^2^ and 0, 5, 10, 15, and 20 min, respectively).

The photodynamic efficacy and dark toxicity of CBT-SiPc against MCF-7 cells were evaluated using CCK-8 assays (Figure 4A). CBT-SiPc was nearly non-cytotoxic in the absence of laser irradiation (Figure 4A), demonstrating its high biocompatibility. Upon irradiation with a 670 nm laser (100 mW/cm^2^) for 10 min, CBT-SiPc showed phototoxicity with an IC_50_ of 4.16 μM in MCF-7 cells. The cell viability also decreased to 33.14% when at a 5 μM concentration of CBT-SiPc after irradiation by a 671 nm laser (100 mW/cm^2^) for 10 min.

Confocal laser scanning microscopy was used to image the ROS generation of CBT-SiPc in MCF-7 cells using DCFH-DA as the probe. As shown in Figure 4B, without irradiation, no green fluorescence was observed in the cells even with CBT-SiPc. Obvious green fluorescence was observed in cells treated with DCFH-DA and CBT-SiPc upon laser irradiation. These results indicated that upon appropriate laser irradiation, CBT-SiPc efficiently generated ROS in MCF-7 cells.

To understand the cell death mechanism induced by CBT-SiPc after PDT treatment, Annexin V-fluorescein isothiocyanate (FITC) and propidium iodide (PI) co-staining was performed (Shobeiri and Sankian, 2022) and analyzed by flow cytometry. As shown in Figure 4C, without light irradiation, most of the cells were viable, with only about 18% of cells showing apoptosis, indicating that CBT-SiPc was non-toxic in the absence of light. Upon light irradiation (671 nm, 100 mW/cm^2^, 10 min), only 17% of cells were viable and 83% were apoptotic. As the percentage of necrotic cells was negligible, we concluded that apoptosis was the major cell death pathway for CBT-SiPc-mediated PDT.

The morphological changes in MCF-7 cells were observed by CLSM (Figure 4D). Before irradiation, CBT-SiPc accumulated in the lysosomes. After irradiation for 10 min, the red fluorescence of CBT-SiPc in the lysosomes had dispersed, the cells shrank and distorted, and the membranes blebbed, indicating that the organization of the lysosomes in the cells was destroyed after PDT. Therefore, the lysosome dysfunction by PDT ultimately led to cell apoptosis.

## 4 Conclusion

This study designed and developed chlorophenyl thiophene silicon (IV) phthalocyanine (CBT-SiPc). Due to the properties of the chlorophenyl thiophene groups, CBT-SiPc selectively targeted lysosomes, as tracked by its two-photon fluorescence. CBT-SiPc also generated ROS in the lysosomes of MCF-7 cells *in situ*, which showed impressive photodynamic activity by inducing cell apoptosis through lysosome dysfunction. These findings demonstrate that CBT-SiPc is a promising photosensitizer for photodynamic therapy.

## Data Availability

The original contributions presented in the study are included in the article/Supplementary Material; further inquiries can be directed to the corresponding authors.

## References

[B1] Agoni C. Ramharack P. Munsamy G. Soliman M. E. S. (2020). Human rhinovirus inhibition through capsid “canyon” perturbation: Structural insights into the role of a novel benzothiophene derivative. Cell Biochem. Biophysics 78 (1), 3–13. 10.1007/s12013-019-00896-z 31834576

[B2] Almeida-Marrero V. Mascaraque M. Jesus Vicente-Arana M. Juarranz A. Torres T. de la Escosura A. (2021). Tuning the nanoaggregates of sialylated biohybrid photosensitizers for intracellular activation of the photodynamic response. Chemistry 27 (37), 9634–9642. 10.1002/chem.202100681 33834569 PMC8360122

[B3] Alshammari G. M. Balakrishnan A. Alshatwi A. A. Al-Khalifa A. (2020). Cucurbita ficifolia fruit extract induces tp53/caspase-mediated apoptosis in MCF-7 breast cancer cells. Biomed. Res. Int. 2020, 3712536. 10.1155/2020/3712536 32685475 PMC7335397

[B4] Bai J. Zhang L. Qian Y. (2021). A near-infrared and lysosomal targeting thiophene-BODIPY photosensitizer: Synthesis and its imaging guided photodynamic therapy of cancer cells. Spectrochim. Acta A Mol. Biomol. Spectrosc. 252, 119512. 10.1016/j.saa.2021.119512 33581575

[B5] Brilkina A. A. Dubasova L. V. Sergeeva E. A. Pospelov A. J. Shilyagina N. Y. Shakhova N. M. (2019). Photobiological properties of phthalocyanine photosensitizers photosens, holosens and phthalosens: A comparative *in vitro* analysis. J. Photochem. Photobiol. B-Biology 191, 128–134. 10.1016/j.jphotobiol.2018.12.020 30616037

[B6] Cai Y. Liang P. Tang Q. Yang X. Si W. Huang W. (2017). Diketopyrrolopyrrole-triphenylamine organic nanoparticles as Multifunctional reagents for photoacoustic imaging-guided photodynamic/photothermal synergistic tumor therapy. ACS Nano 11 (1), 1054–1063. 10.1021/acsnano.6b07927 28033465

[B7] Chen K. Li X. Yu X. Zhang T. Ye Q. Xiao W. (2019). Copper-cysteamine nanoparticles encapsulating fluorocoumarin silicon(IV) phthalocyanines: Synthesis, characterization, and photophysical properties. J. Coord. Chem. 72 (22-24), 3589–3601. 10.1080/00958972.2019.1703184

[B8] Chen X. Ye Q. Ma D. Chen J. Wang Y. Yang H. (2018a). Gold nanoparticles-pyrrolidinonyl metal phthalocyanine nanoconjugates: Synthesis and photophysical properties. J. Luminescence 195, 348–355. 10.1016/j.jlumin.2017.11.047

[B9] Chen Y. Liu J. Song M. Jiang L. Liu L. Liu Y. (2018b). Insights into the binding mechanism of BODIPY-based photosensitizers to human serum albumin: A combined experimental and computational study. Spectrochim. Acta A Mol. Biomol. Spectrosc. 203, 158–165. 10.1016/j.saa.2018.05.103 29864639

[B10] D'Alessandro S. Priefer R. (2020). Non-porphyrin dyes used as photosensitizers in photodynamic therapy. J. Drug Deliv. Sci. Technol. 60, 101979. 10.1016/j.jddst.2020.101979

[B11] Darwish W. (2020). Polymers for enhanced photodynamic cancer therapy: Phthalocyanines as a photosensitzer model. Polym. Adv. Technol. 32 (3), 919–930. 10.1002/pat.5154

[B12] Davies D. A. Schnik C. Silver J. Sosa-Sanchez J. L. Riby P. G. (2012). A high-yield microwave heating method for the preparation of (phthalocyaninato)bis(chloro)silicon(IV). J. Porphyr. Phthalocyanines 05 (04), 376–380. 10.1002/jpp.293

[B13] Ding G.-b. Liu H.-y. Wang Y. Lü Y.-y. Wu Y. Guo Y. (2013). Fabrication of a magnetite nanoparticle-loaded polymeric nanoplatform for magnetically guided drug delivery. Chem. Res. Chin. Univ. 29 (1), 103–109. 10.1007/s40242-013-2134-7

[B14] Ding Y. F. Li S. Liang L. Huang Q. Yuwen L. Yang W. (2018). Highly Biocompatible Chlorin e6-Loaded Chitosan Nanoparticles for Improved Photodynamic Cancer Therapy. ACS Appl. Mater Interfaces 10 (12), 9980–9987. 10.1021/acsami.8b01522 29498260

[B15] Elkanzi N. A. A. (2018). Short review on the synthesis of thiophene, pyrazole, and thiazole derivatives. Pyrazole, Thiazole Deriv. 65 (2), 189–204. 10.1002/jccs.201700207

[B16] Forteath S. Antunes E. Chidawanyika W. Nyokong T. (2012). Unquenched fluorescence lifetime for β-phenylthio substituted zinc phthalocyanine upon conjugation to gold nanoparticles. Polyhedron 34 (1), 114–120. 10.1016/j.poly.2011.12.015

[B17] Galstyan A. (2021). Turning photons into drugs: Phthalocyanine-based photosensitizers as efficient photoantimicrobials. Chemistry 27 (6), 1903–1920. 10.1002/chem.202002703 32677718 PMC7894475

[B18] Gao D. Lo P.-C. (2018). Polymeric micelles encapsulating pH-responsive doxorubicin prodrug and glutathione-activated zinc(II) phthalocyanine for combined chemotherapy and photodynamic therapy. J. Control. Release 282, 46–61. 10.1016/j.jconrel.2018.04.030 29673646

[B19] Gleiter R. Haberhauer G. Werz D. B. Rominger F. Bleiholder C. (2018). From noncovalent chalcogen-chalcogen interactions to supramolecular aggregates: Experiments and calculations. Chem. Rev. 118 (4), 2010–2041. 10.1021/acs.chemrev.7b00449 29420879

[B20] Karaoğlu H. R. P. Yenilmez H. Y. Koçak M. B. (2018). Phthalocyanines formed from several precursors: Synthesis, characterization, and comparative fluorescence and quinone quenching. J. Coord. Chem. 71 (15), 2340–2357. 10.1080/00958972.2018.1471686

[B21] Kim J. Santos O. A. Park J.-H. (2014). Selective photosensitizer delivery into plasma membrane for effective photodynamic therapy. J. Control. Release 191, 98–104. 10.1016/j.jconrel.2014.05.049 24892975

[B22] Li X. Zheng B.-D. Peng X.-H. Li S.-Z. Ying J.-W. Zhao Y. (2019). Phthalocyanines as medicinal photosensitizers: Developments in the last five years. Coord. Chem. Rev. 379, 147–160. 10.1016/j.ccr.2017.08.003

[B23] Liu C. Yan H. Wu J. Wang Z. He S. Zhao L. (2022). Lysosomes-targeting near-infrared fluorescent probe for the detection of pH in living cells. Spectrochim. Acta A Mol. Biomol. Spectrosc. 278, 121368. 10.1016/j.saa.2022.121368 35569197

[B24] Liu G. Ma S. Li S. Cheng R. Meng F. Liu H. (2010). The highly efficient delivery of exogenous proteins into cells mediated by biodegradable chimaeric polymersomes. Biomaterials 31 (29), 7575–7585. 10.1016/j.biomaterials.2010.06.021 20599266

[B25] Liu Q. C. Li J. Liu C. He S. Zhao L. C. Zeng X. S. (2021). Developing a NIR emitting benzothiazolium-thioxanthene dye and its application for the design of lysosomes-targeting palladium(II) probe. Dyes Pigments 196, 109796. 10.1016/j.dyepig.2021.109796

[B26] Luo T. Wang D. Liu L. Zhang Y. Han C. Xie Y. (2021). Switching reactive oxygen species into reactive nitrogen species by photocleaved O2-released nanoplatforms favors hypoxic tumor repression. Adv. Sci. 8 (19), 2101065. 10.1002/advs.202101065 PMC8498884 34369112

[B27] Meng Y. Bai X. Huang Y. He L. Zhang Z. Li X. (2019). Basic fibroblast growth factor signalling regulates cancer stem cells in lung cancer A549 cells. J. Pharm. Pharmacol. 71 (9), 1412–1420. 10.1111/jphp.13136 31282010

[B28] Ming L. Cheng K. Chen Y. Yang R. Chen D. (2021). Enhancement of tumor lethality of ROS in photodynamic therapy. Cancer Med. 10 (1), 257–268. 10.1002/cam4.3592 33141513 PMC7826450

[B29] Miretti M. Prucca G. C. Tempesti C. T. Baumgartner T. M. (2021). Current phthalocyanines delivery systems in photodynamic therapy: An updated review. Curr. Med. Chem. 28 (26), 5339–5367. 10.2174/0929867328666210208111234 33557727

[B30] Mishra R. Kumar N. Mishra I. Sachan N. (2020). A review on anticancer activities of thiophene and its analogs. Mini-Reviews Med. Chem. 20 (19), 1944–1965. 10.2174/1389557520666200715104555 32669077

[B31] Mohareb R. M. Abdallah A. E. M. Helal M. H. E. Shaloof S. M. H. (2016). Synthesis and structure elucidation of some novel thiophene and benzothiophene derivatives as cytotoxic agents %J. Acta Pharm. 66(1), 53–68. 10.1515/acph-2016-0005 26959543

[B32] Özdemir A. Turanli S. Çalişkan B. Arka M. Banoglu E. (2020). Evaluation of cytotoxic activity of new benzimidazole-piperazine hybrids against human MCF-7 and A549 cancer cells. Pharm. Chem. J. 53 (11), 1036–1046. 10.1007/s11094-020-02119-9

[B33] Simoes J. C. S. Sarpaki S. Papadimitroulas P. Therrien B. Loudos G. (2020). Conjugated photosensitizers for imaging and PDT in cancer research. J. Med. Chem. 63 (23), 14119–14150. 10.1021/acs.jmedchem.0c00047 32990442

[B34] Soriano J. Villanueva A. Stockert J. C. Cañete M. (2014). Regulated necrosis in HeLa cells induced by ZnPc photodynamic treatment: A new nuclear morphology. A New Nucl. Morphol. 15 (12), 22772–22785. 10.3390/ijms151222772 PMC4284736 25501332

[B35] Valli F. García Vior M. C. Roguin L. P. Marino J. (2019). Oxidative stress generated by irradiation of a zinc(II) phthalocyanine induces a dual apoptotic and necrotic response in melanoma cells. Apoptosis 24 (1), 119–134. 10.1007/s10495-018-01512-w 30603830

[B36] Wang Y. Xia C. Chen W. Chen Y. Wang Y. Li T. (2014). Autoregulatory feedback mechanism of P38mapk/caspase-8 in photodynamic therapy-hydrophilic/lipophilic tetra-α-(4-carboxyphenoxy) phthalocyanine zinc-induced apoptosis of human hepatocellular carcinoma bel-7402 cells. Int. J. Photoenergy 2014, 1–9. 10.1155/2014/163813

[B37] Yu L. Wang Q. Yeung K.-W. Fong W.-P. Lo P.-C. (2018). A biotinylated and endoplasmic reticulum-targeted glutathione-responsive zinc(II) phthalocyanine for targeted photodynamic therapy. Chem. Asian J. 13 (22), 3509–3517. 10.1002/asia.201800852 29956487

[B38] Yu S. J. Liu H. C. Ling-Ling E. Wang D. S. Zhu G. X. (2012). Proliferation and differentiation of osteoblasts from the mandible of osteoporotic rats. Exp. Biol. Med. (Maywood) 237 (4), 395–406. 10.1258/ebm.2011.011217 22550338

[B39] Zhang X. F. Lin Y. Guo W. Zhu J. (2014). Spectroscopic insights on imidazole substituted phthalocyanine photosensitizers: Fluorescence properties, triplet state and singlet oxygen generation. Spectrochim. Acta A Mol. Biomol. Spectrosc. 133, 752–758. 10.1016/j.saa.2014.06.063 24997445

[B40] Zou J. Zhang Y. Sun J. Wang X. Tu H. Geng S. (2017). Deoxyelephantopin induces reactive oxygen species-mediated apoptosis and autophagy in human osteosarcoma cells. Cell Physiol. Biochem. 42 (5), 1812–1821. 10.1159/000479537 28750364

