# Supplementary material for: Chlorophenyl thiophene silicon phthalocyanine: Synthesis, two-photon bioimaging-guided lysosome target, and in vitro photodynamic efficacy
Source: Front Pharmacol. 2023 Apr 13;14:1168393. doi: 10.3389/fphar.2023.1168393 (PMC10133558; doi:10.3389/fphar.2023.1168393)
Supplement: Supplementary file 1 [file DataSheet1.docx]

Supplementary Material

**Chlorophenyl thiophene silicon phthalocyanine: synthesis, two-photon bioimaging-guided lysosomes target and in vitro photodynamic efficacy**

**1. Photophysical properties**

The UV–Vis and fluorescence spectra of CBT were at 25 ℃ with N, N-dimethylformamide (DMF) as solvent.

Fluorescence quantum yield (Ф_F_) of CBT were determined to the literature(Ogunsipe et al., 2004; Forteath et al., 2012; Demirbaş, 2020) by using Eq.S1.

$\Phi_{F}=\text{Ф}\text{F }_{\text{(std)}}\text{∙F∙}\text{A}_{\text{std}}\text{/ (}\text{F}_{\text{std}}\text{∙A)}$ $\text{Ф}\text{F=}\frac{\text{Ф}\text{F }_{\text{(std)}}\text{∙F∙}\text{A}_{\text{std}}}{\text{F}_{\text{std}}\text{∙A}}$ $\boldsymbol{\Phi}_{\boldsymbol{F}}\boldsymbol{=}\text{Ф}\text{F }_{\text{(std)}}\text{∙F∙}\text{A}_{\text{std}}\text{/ (}\text{F}_{\text{std}}\text{∙A)}$ $\Phi_{F}$ (Eq. S1)


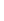


Unsubstituted zinc phthalocyanine (n-ZnPc, ФF = 0.28) in DMF was employed as the standard. Besides, F and FStd are the integral areas of emission curves of the CBT and n-ZnPc, respectively. AStd is the absorbance at the excitation wavelength of standard n-ZnPc; A is the absorbance of the phthalocyanine at the excitation wavelength.

The fluorescence lifetime (τ_s_) was measured using the standard technique of time correlated single photon counting (TCSPC)(Lacey and Phillips, 2002; Pişkin, 2016), and the fluorescence decay profiles were found to fit to the monoexponential function (Eq.2).

$\text{f}\left( \text{t} \right)\text{=A+}{\text{B}_{\text{1}}\text{e}}^{\text{-t/}\text{τ1}}\text{+}{\text{B}_{\text{2}}\text{e}}^{\text{-t/}\text{τ2}}\text{+}{\text{B}_{\text{3}}\text{e}}^{\text{-t/}\text{τ3}}$ (Eq.2)

1,3-diphenylisobenzofuran (DPBF) was employed to determine the singlet oxygen quantum yield with n-ZnPc (ΦΔref=0.56) in DMF as the standard. ФΔ was calculated by the relative method(Çakır et al., 2014) according to Eq. 6 and Eq. 7.

$\boldsymbol{Ф}_{\boldsymbol{\Delta}}\boldsymbol{=}{\boldsymbol{Ф}_{\boldsymbol{\Delta}}^{\boldsymbol{ref}}}\frac{\boldsymbol{k}}{\boldsymbol{k}^{\boldsymbol{ref}}} \frac{\boldsymbol{I}_{\boldsymbol{a}}^{\boldsymbol{ref}}}{\boldsymbol{I}_{\boldsymbol{a}}}$ (Eq. 6)

$\frac{\boldsymbol{I}_{\boldsymbol{a}}^{\boldsymbol{ref}}}{\boldsymbol{I}_{\boldsymbol{a}}}\boldsymbol{=}\frac{\boldsymbol{1}\boldsymbol{-}\boldsymbol{10}^{\boldsymbol{-}\boldsymbol{A}_{\boldsymbol{670}}^{\boldsymbol{ref}}}}{\boldsymbol{1}\boldsymbol{-}\boldsymbol{10}^{\boldsymbol{-}\boldsymbol{A}_{\boldsymbol{670}}}}$ (Eq. 7)

Among them, K is the photobleaching constant of DPBF/ABDA. Kref is the photobleaching constant of DPBF/ABDA in n-ZnPc; Ia and Iref a indicate the absorption rates of the samples and n-ZnPc at the irradiation wavelength of 670 nm, respectively. A670 and Aref 670 are the absorbance of the samples and n-ZnPc at the irradiation wavelength of 670 nm, respectively.

**2. Characterization**

**2.1 Characterization of CBT-OH**


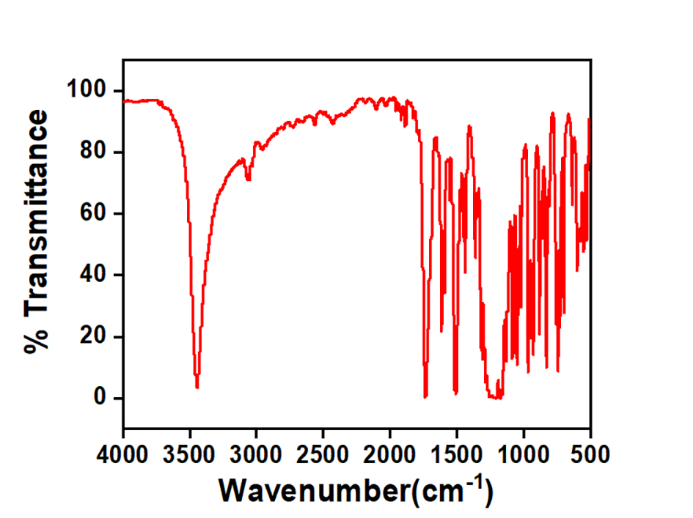


**Fig. S1** The FT-IR spectrum of CBT-OH


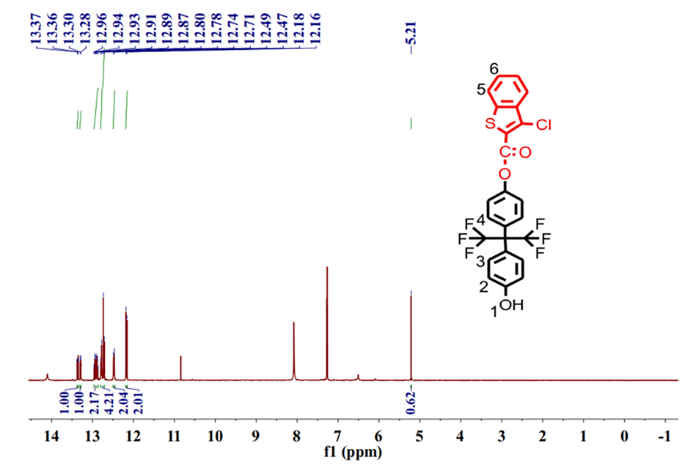


**Fig. S2** The ^1^HNMR spectrum of CBT-OH (400M Hz, CDCl_3_)


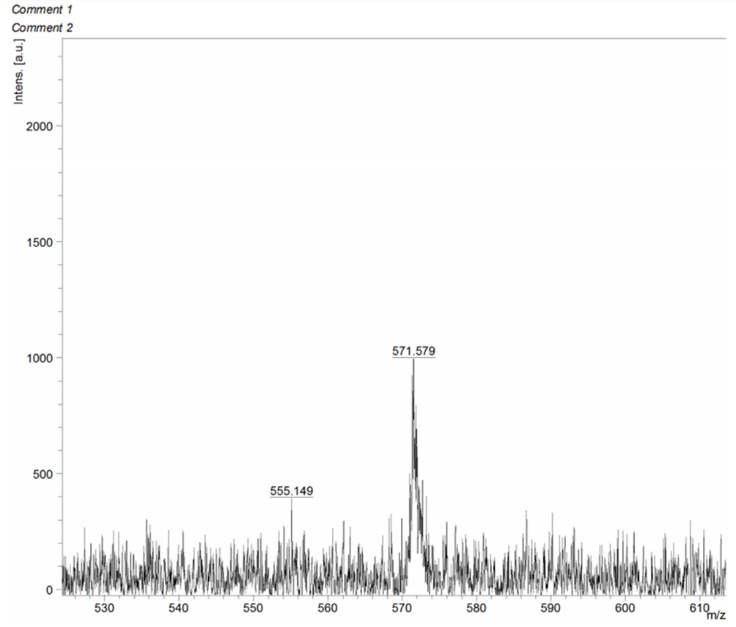


**Fig. S3** The ESI-MS spectrum of CBT-OH

**2.2 Characterization of CBT-SiPc**


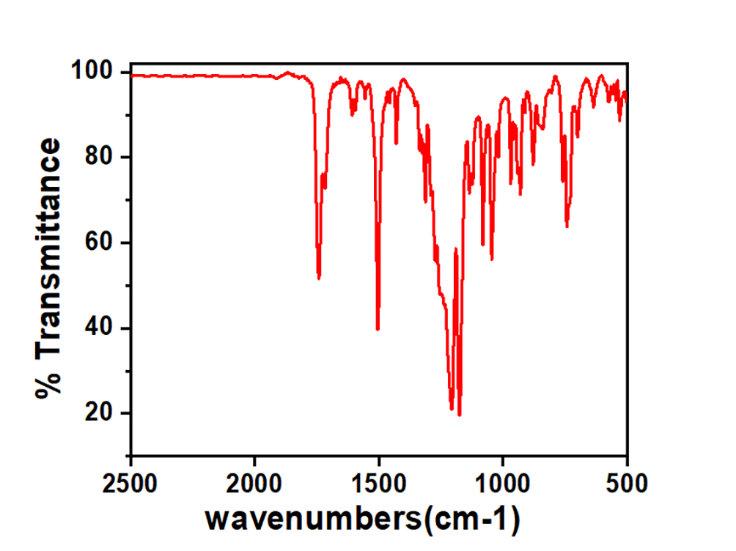


**Fig. S4** The FT-IR spectrum of CBT-SiPc


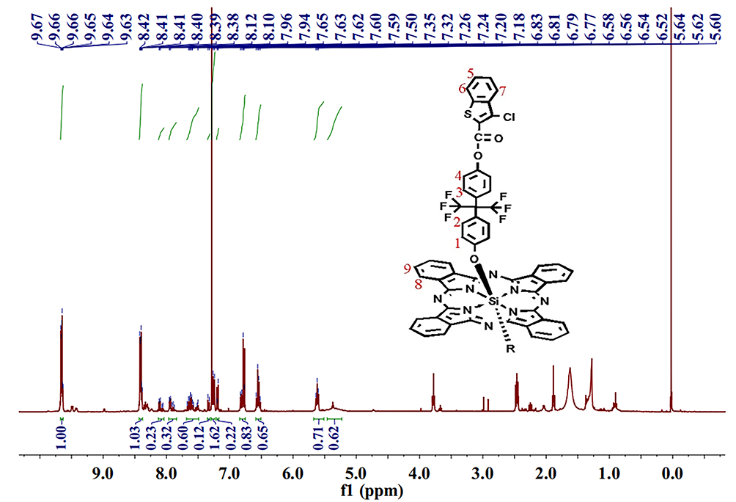


**Fig. S5** The ^1^HNMR spectrum of CBT-SiPc (400M Hz, CDCl_3_)

**
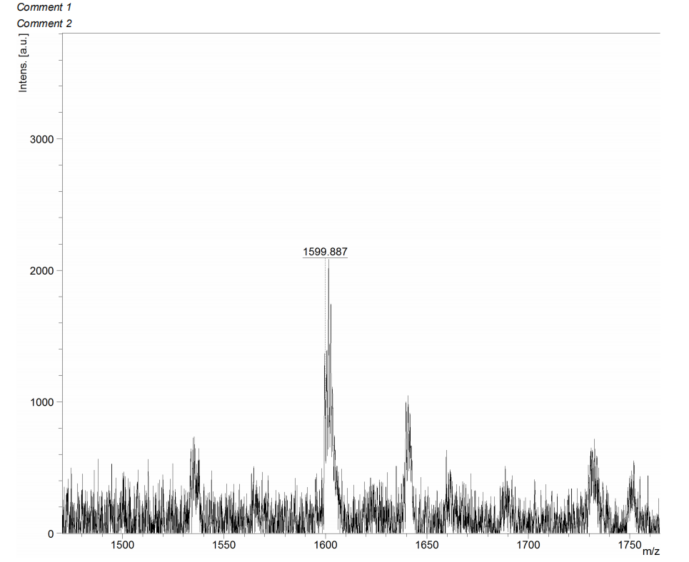
**

**Fig. S6** The ESI-MS spectrum of CBT-SiPc


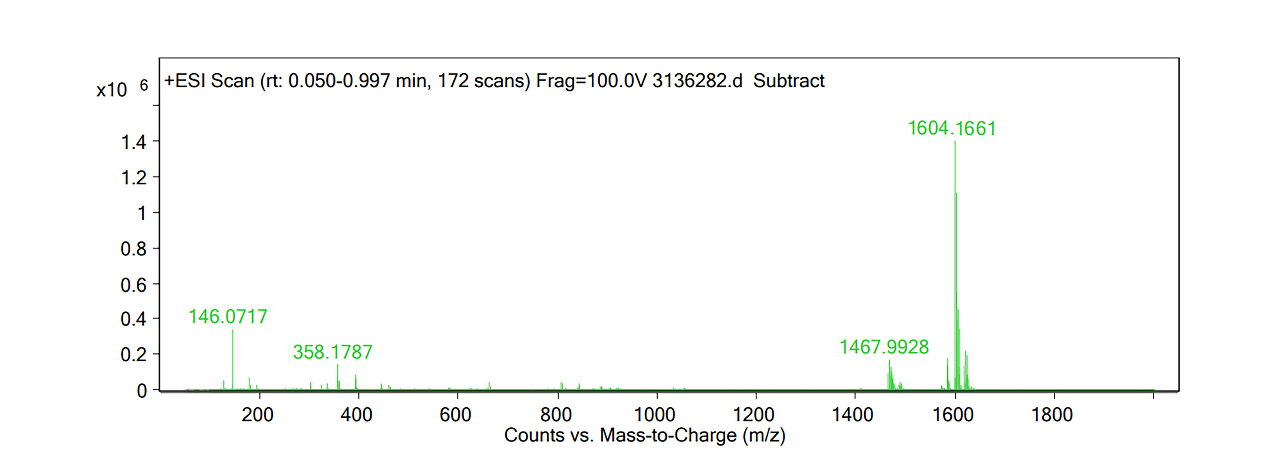


**Fig. S7** HRMS of CBT-SiPc


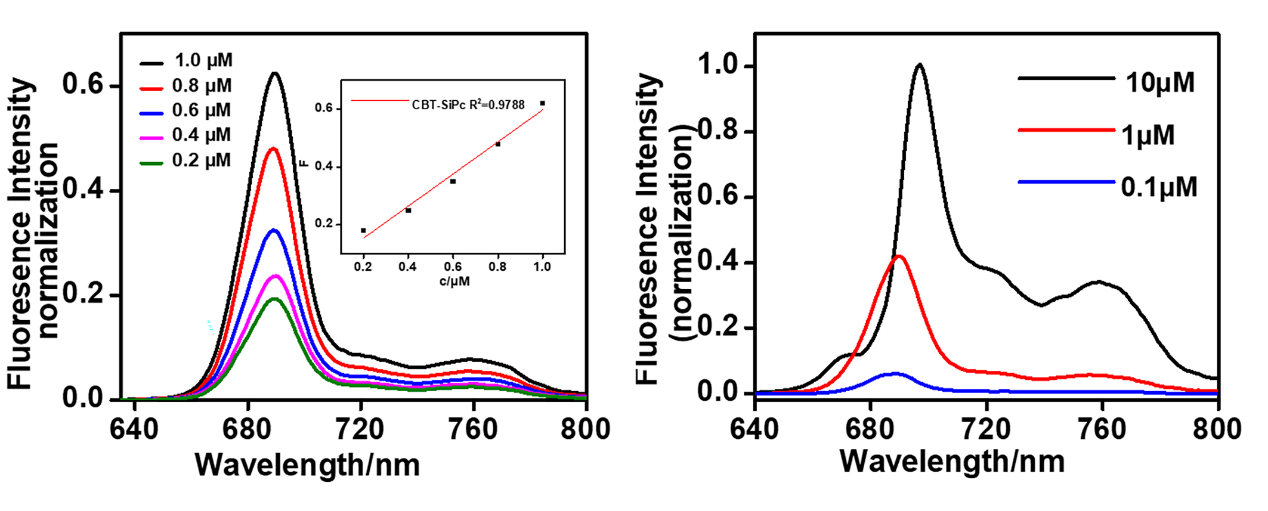


**Fig. S8** Fluorescence spectra of CBT-SiPc in DMF (The inset shows the fluorescence intensity at 689 nm, as a function of its concentration λ_ex_=615 nm)


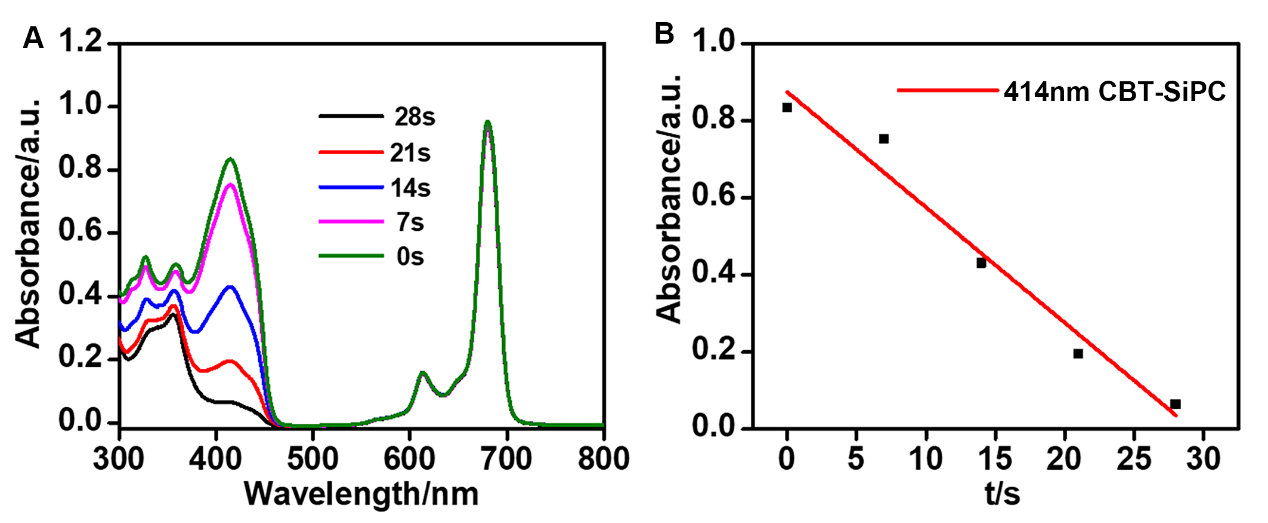


**Fig. S9** A typical absorption spectral change for determination of singlet oxygen quantum yield of CBT-SiPc in DMF using DPBF as a singlet oxygen quencher under irradiation at 670 nm (35 mW/cm^2^) (**Fig. S8A**) The linear plot of absorbance at 414 nm against irradiation time (**Fig. S8B**)


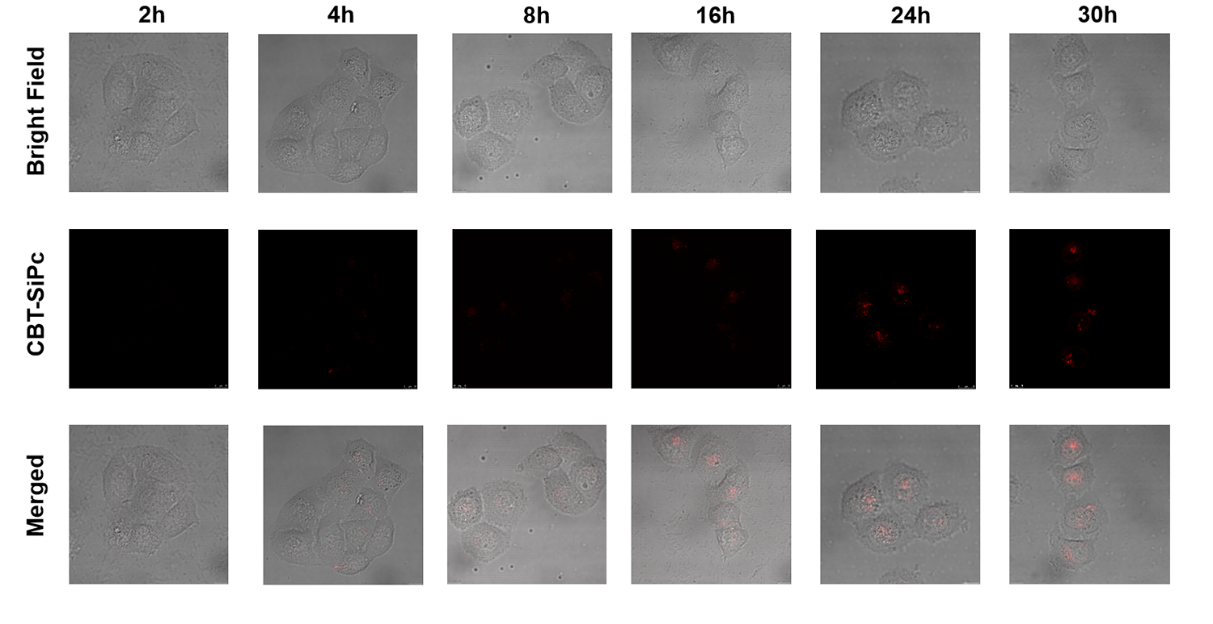


**Fig. S10** Two-photon fluorescence images of CBT-SiPc (2 uM) incubated with MCF-7 breast cancer with different time (2,4,8,16,24,30 h). (Two-photon fluorescence images of CBT-SiPc in MCF-7 breast cancer cells (2 µM, red fluorescence, excited by 860 nm femtosecond laser and its fluorescence was monitored at 650-700 nm)


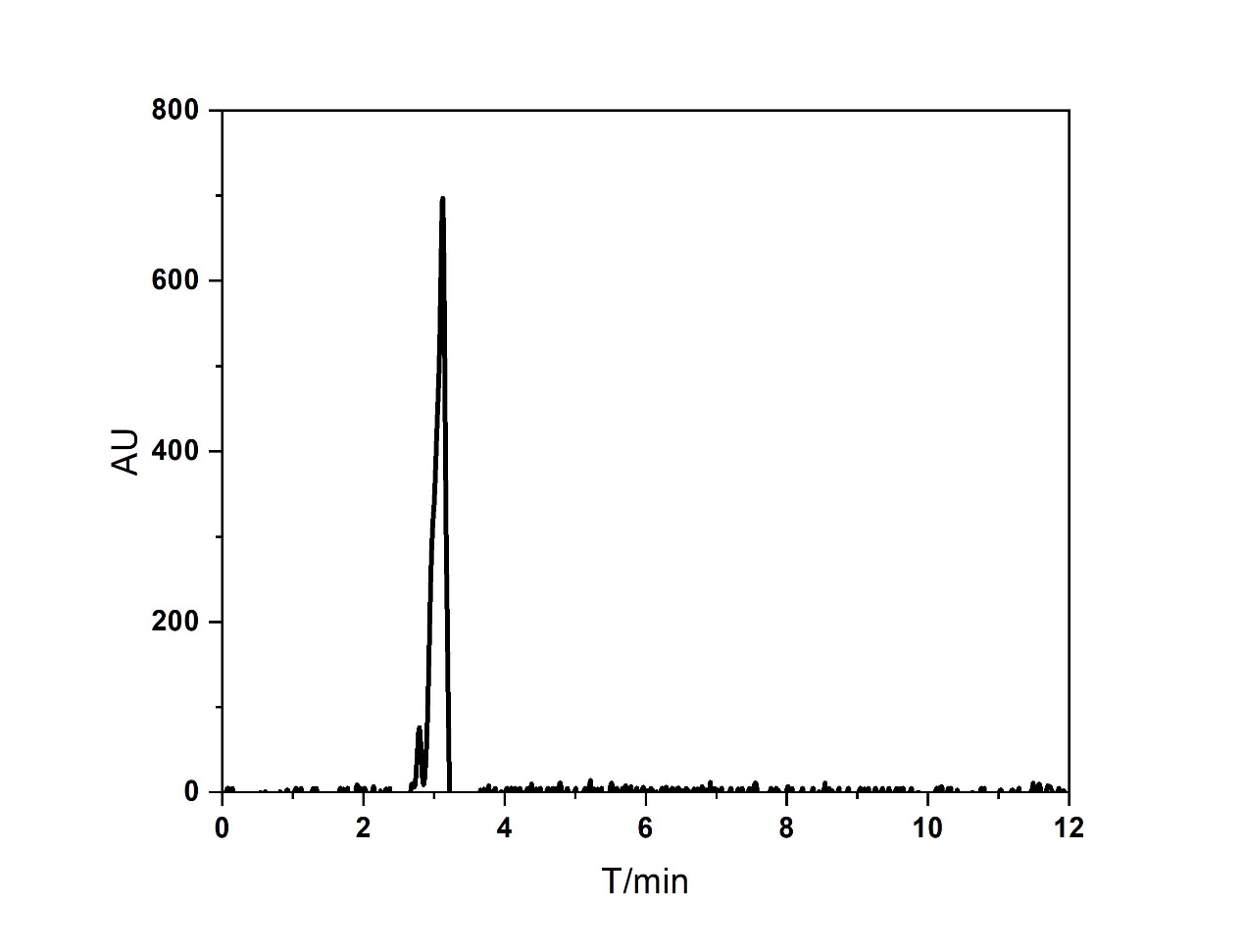


**Fig. S11 The HPLC spectrum of CBT-SiPc.**

Reference

Çakır, V., Çakır, D., Pişkin, M., Durmuş, M., and Bıyıklıoğlu, Z. (2014). Water soluble peripheral and non-peripheral tetrasubstituted zinc phthalocyanines: Synthesis, photochemistry and bovine serum albumin binding behavior. *Journal of Luminescence* 154**,** 274-284. doi: 10.1016/j.jlumin.2014.04.030.

Demirbaş, Ü. (2020). Novel peripherally tetra substituted phthalocyanines: Synthesis, characterization, photophysical and photochemical properties. *Journal of Molecular Structure* 1211. doi: 10.1016/j.molstruc.2020.128082.

Forteath, S., Antunes, E., Chidawanyika, W., and Nyokong, T. (2012). Unquenched fluorescence lifetime for β-phenylthio substituted zinc phthalocyanine upon conjugation to gold nanoparticles. *Polyhedron* 34(1)**,** 114-120. doi: 10.1016/j.poly.2011.12.015.

Lacey, J.A., and Phillips, D. (2002). Fluorescence lifetime measurements of disulfonated aluminium phthalocyanine in the presence of microbial cells. *Photochem Photobiol Sci* 1(6)**,** 378-383. doi: 10.1039/b108831a.

Ogunsipe, A., Chen, J.-Y., and Nyokong, T. (2004). Photophysical and photochemical studies of zinc(ii) phthalocyanine derivatives—effects of substituents and solvents. *New J. Chem.* 28(7)**,** 822-827. doi: 10.1039/b315319c.

Pişkin, M. (2016). The novel 2,6-dimethoxyphenoxy substituted phthalocyanine dyes having high singlet oxygen quantum yields. *Polyhedron* 104**,** 17-24. doi: 10.1016/j.poly.2015.11.017.
